# Impact of Daily Uplifts and Hassles on Daily Awareness of Age-Related Changes in Late Life

**DOI:** 10.1093/geroni/igaf122.1493

**Published:** 2025-12-31

**Authors:** Yiwen Wu, Hongmei Lin, Helene Fung

**Affiliations:** The Chinese University of Hong Kong, Hong Kong, China (People’s Republic); The Chinese University of Hong Kong, Hong Kong, China (People’s Republic); The Chinese University of Hong Kong, Hong Kong, China (People’s Republic)

## Abstract

**Background:**

Awareness of Age-Related Changes (AARC), which refers to individuals’ awareness of age-related gains and losses, is a crucial contributor to subjective aging experiences. While previous research has focused on exploring factors related to AARC at the trait level, little is known about the contributors to AARC at the daily level.

**Purpose:**

This study aims to investigate the relationship between daily uplifts and hassles and daily AARC and to explore potential moderators.

**Methods:**

A total of N = 286 older participants, aged 50 to 80 years (M = 60.8, SD = 6.1), were recruited in Hong Kong. Participants reported their daily AARC, as well as daily hassles and uplifts, every night over 14 consecutive days, along with baseline and demographic measures.

**Results:**

The results showed that participants were not only more aware of age-related losses on days with more daily hassles but also, surprisingly, on days with more daily uplifts. This suggests that even positive life experiences might become sources of awareness related to loss in old age. Additionally, trait-level non-essentialist beliefs about aging and sense of mattering may serve as protective factors, buffering the impact of daily experiences on age-related loss awareness. Finally, neither daily uplifts nor daily hassles were significantly related to AARC gains at the daily level.

**Conclusion:**

This study reveals the complex dynamics between daily events and subjective aging. Moreover, non-essentialist beliefs about aging and a sense of mattering may serve as protective factors, buffering the impact of daily experiences on age-related loss awareness.

